# Measurement and Clinical Significance of Lipid Peroxidation as a Biomarker of Oxidative Stress: Oxidative Stress in Diabetes, Atherosclerosis, and Chronic Inflammation

**DOI:** 10.3390/antiox8030072

**Published:** 2019-03-25

**Authors:** Fumiaki Ito, Yoko Sono, Tomoyuki Ito

**Affiliations:** 1 The Institute of Prophylactic Pharmacology, Shinagawa, Tokyo 140-0001, Japan; 2 R&D Department, Sunstar Inc., Takatsuki, Osaka 569-1195, Japan; yoko.sono@jp.sunstar.com; 3 Physical Medicine and Rehabilitation, Tanabe Memorial Hospital, Kyotanabe-City, Kyoto 610-0331, Japan; rinito@par.odn.ne.jp; 4 Department of Rehabilitation Medicine, Kyoto Prefectural University of Medicine, Kyoto 602-8566, Japan

**Keywords:** oxidative stress, oxidative stress biomarkers, lipid peroxidation, 8-isoprostaglandin F_2_α, malondialdehyde, Fe-ROMs test, diabetes mellitus, endothelial dysfunction, atherosclerosis, cardiovascular disease, chronic inflammation, oxidized high-density lipoprotein

## Abstract

Endothelial dysfunction is one of the initial steps in the pathogenesis of atherosclerosis and development of cardiovascular disease in patients with diabetes mellitus. Several risk factors are associated with endothelial dysfunction and atherosclerosis, such as hypertension, dyslipidaemia, inflammation, oxidative stress, and advanced glycation-end products. Among these risk factors, oxidative stress is the largest contributor to the formation of atherosclerotic plaques. Measurement of reactive oxygen species (ROS) is still difficult, and assays for the measurement of ROS have failed to show a consistent correlation between pathological states and oxidative stress. To solve this problem, this review summarizes the current knowledge on biomarkers of oxidative stress, especially lipid peroxidation, and discusses the roles of oxidative stress, as measured by indices of lipid peroxidation, in diabetes mellitus, atherosclerosis, and chronic inflammation.

## 1. Introduction-What is Oxidative Stress?

Oxidative stress can be caused by excess reactive oxygen species (ROS) and reactive nitrogen species (RNS). ROS include superoxide (O_2_•^−^), hydrogen peroxide (H_2_O_2_), hydroxyl radical (•OH), and singlet oxygen (^1^O_2_). Nitrogen-containing oxidants, such as nitric oxide (NO•), peroxynitrite (ONOO^−^), and nitrogen dioxide (NO_2_), are known as RNS. NO• is relatively unreactive, whereas ONOO^−^, the product of the diffusion-controlled reaction of NO• and O_2_•^−^, is a powerful oxidant [1]. The control of ROS and RNS levels is provided not only via their production, but also via elimination. Imbalance between the production and elimination in favor of the first, with certain consequences for cell physiology, has been called “oxidative stress” [2]. Accumulation of reactive oxygen and nitrogen species leads to oxidative damage to virtually all molecules. These reactive species are not necessarily a threat to the body under normal physiological conditions [3,4], but when the body fails to remove them to a certain degree, oxidative stress stimulates the formation of atherosclerotic plaques and increases the risk of coronary artery disease, type 2 diabetes mellitus (T2DM), and atherosclerosis [5,6].

Despite the accumulated knowledge on the roles of oxidative stress in many serious pathophysiological processes, measurement of ROS is still difficult. Electron spin resonance has been recognized as the most powerful technique for the detection of ROS in the form of free radicals. However, ROS are short lived and do not accumulate to sufficiently high levels to be measured.

ROS oxidize various biological macromolecules, such as proteins, lipids, and nucleic acids, thus causing structural and functional changes in these molecules. Lipid oxidation generates hydroperoxides, which subsequently undergo fragmentation to produce a broad range of reactive intermediates, such as prostaglandin F2α isomer F_2_-isoprostanes (F_2_-IsoPs), and malondialdehyde (MDA) [7,8,9]. Because lipids in biological membranes and lipoproteins are major peroxidation targets, assays for lipid peroxidation are commonly used for estimation of the oxidative status. Another approach to measuring oxidative damage is by the modifications of proteins and nucleic acids. The common assays to assess such protein modifications are to measure the nitration of protein tyrosine residues and the carbonyl groups of the oxidized proteins. 3-Nitrotyrosine content is assessed by western blotting, high-performance liquid chromatography (HPLC), gas chromatography-mass spectrometry (GC/MS), and enzyme-linked immunosorbent assay (ELISA) [10,11]. Among these analytical techniques, ELISA is most commonly used to measure 3-nitrotyrosine, because it is a low-cost, simple, and convenient assay. 8-Hydroxy-2′-deoxyguanosine (8-OHdG) and 8-hydroxyguanosine (8-OHG) are biomarkers of oxidative damage of nucleic acids, which can be assessed by ELISA, as well as by direct methods such as HPLC and GC/MS [12]. Attempts to evaluate the relationship between these various assays of oxidative stress found good correlations between the concentrations of several oxidation products, including lipid hydroperoxides, conjugated dienes, MDA, F_2_-IsoPs, glutathione (GSH), and protein carbonyls, but not with other criteria of oxidative stress, such as the concentration of oxidized nucleic acids [12]. 

In addition to oxidatively-modified lipids, proteins, and nucleic acids, several other parameters, such as activities of antioxidant enzymes, are used as biomarkers of oxidative stress. The use of the different parameters for the assessment of oxidative stress doesn’t always come to the same conclusion. Hence, oxidative stress cannot be defined by any universal index. The lack of correlation between different parameters is partly explained by the different kinetics of production and elimination of the biomarkers [13]. Among many parameters, the levels of oxidatively-modified components are commonly accepted markers of oxidative stress, because they increase at an earlier time than others under stressful conditions, and their increase occurs dependent on the dose or concentration of effector inducing oxidative stress [2]. Moreover, time-dependent monitoring of the levels of oxidatively-modified components is relatively easy.

Lipid peroxidation is thought to be a useful target for assessment of oxidative stress because the hydroxyl radical is the most reactive form of ROS and can initiate lipid peroxidation by attacking polyunsaturated fatty acids (PUFA) [14]. This review will focus on lipid peroxidation as biomarkers of oxidative stress. The latter part of the review will deal with the roles of oxidative stress, as measured by indices of lipid peroxidation, in various pathophysiological processes, including diabetes mellitus, endothelial dysfunction, and chronic inflammation. All of the assay methods for measurement of lipid peroxidation use a biological fluid (blood, serum, plasma, and urine). Therefore, it is expected that oxidative stress in the tested fluid, as measured by indices of lipid peroxidation, will reflect the state of oxidative stress in relevant tissues. 

## 2. Lipid Peroxidation

**Figure 1:**
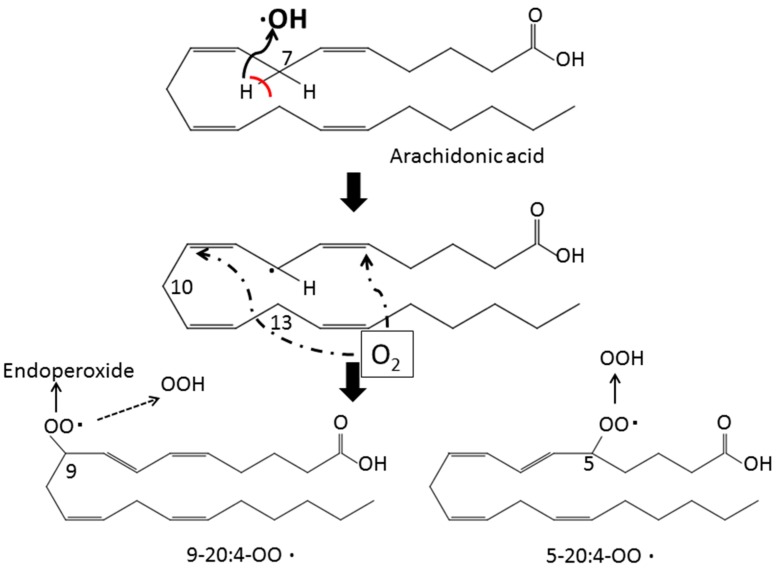
Formation of arachidonic acid hydroperoxides. Abstraction of the *bis*-allylic hydrogen leads to the attack of oxygen to either of two positions and the formation of arachidonic acid peroxyl radical (LOO•). The peroxyradical (5-20:4-OO•) is converted to arachidonic acid hydroperoxides (5-20:4-OOH) and propagates the reaction as the peroxyl radical abstracts a second hydrogen atom from the polyunsaturated fatty acid (PUFA). On the other hand, the peroxyradical (9-20:4-OO•) shows a tendency to generate its corresponding endoperoxide rather than arachidonic acid hydroperoxide (9-20:4-OOH).

**Figure 2:**
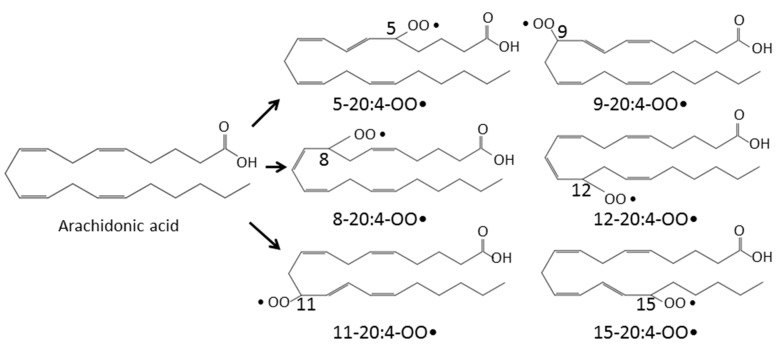
Formation of various arachidonic acid hydroperoxides. As described in Figure 1, abstraction of the *bis*-allylic hydrogen attached to the –C– atom at position 7 leads to the formation of arachidonic acid peroxides (5-20:4-OO• or 9-20:4-OO•). Abstraction of the *bis*-allylic hydrogen at position 10 and 13 generates arachidonic acid hydroperoxides (8-20:4-OO• or 12-20:4-OO•) and hydroperoxides (11-20:4-OO• or 15-20:4-OO•), respectively. Arachidonic acid hydroperoxides (5-20:4-OOH and 15-20:4-OOH) are generated from arachidonic acid peroxides (5-20:4-OO• and 15-20:4-OO•), respectively. On the other hand, arachidonic acid peroxides (8-20:4-OO•, 9-20:4-OO•, 11-20:4-OOH and 12-20:4-OO•) are converted to their corresponding endoperoxides, respectively.

**Figure 3:**
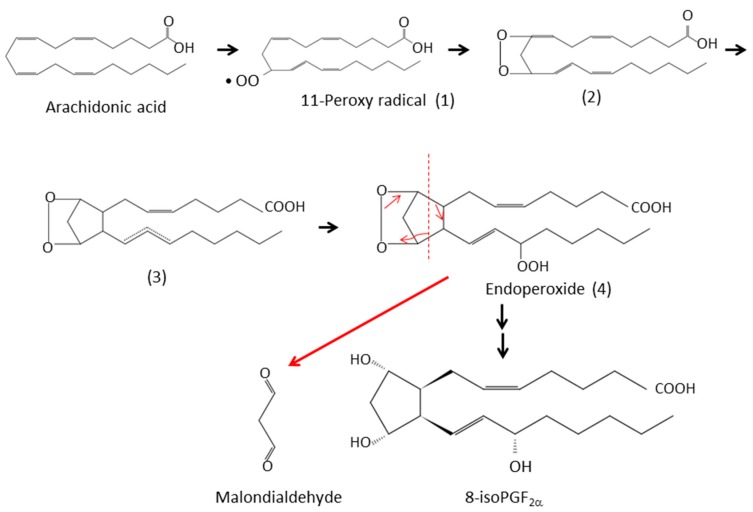
Formation of 8-isoprostaglandin F_2_α (8-isoPGF_2_α) and malondialdehyde from arachidonic acid. 11-Peroxy radical (11-20:4-OO•) is generated from arachidonic acid and then undergoes subsequent cyclization to generate the bicyclic endoperoxide (4). The unstable bicycloendoperoxide (4) is then reduced to the 8-isoPGF_2_α or cleaved to produce malondialdehyde (indicated in red).

Hydrogen peroxide (H_2_O_2_) is produced from superoxide anion (O_2_•^−^) by superoxide dismutase (SOD) through a dismutation reaction. It is the least reactive molecule among ROS and is stable under physiological pH. The hydroxyl radical (•OH), the most reactive and hazardous radical, is formed from H_2_O_2_ in the presence of metal ions, and it has a very short in vivo half-life. The hydroxyl radical plays an important role in the reactions of lipid peroxidation. It oxidizes lipids containing carbon-carbon double bonds, especially polyunsaturated fatty acids (PUFAs). In PUFAs, the –C=C– units are separated by a single-bonded –C– atom. The hydrogen atoms attached to these –C– atoms (*bis*-allylic hydrogens) are susceptible to attack by hydroxyl radical. Therefore, PUFAs, such as arachidonic (20:4) and docosahexaenoic acid (22:6), are highly peroxidizable fatty acids. Arachidonic acid has a 20-carbon chain as a backbone and four cis-double bonds at the C5, C8, C11, and C14 positions and consequently three *bis*-allylic hydrogens (Figure 1). The abstraction of a *bis*-allylic hydrogen attached to a –C– atom at position 7 and the subsequent insertion of oxygen results in 5-20:4-OOH or 9-20:4-OOH. Similarly, the hydrogen abstraction at C10 and C13 generates 8-20:4-OO• or 12-20:4-OO• and 11-20:4-OO• or 15-20:4-OO•, respectively (Figure 2). Among these arachidonic acid peroxides, 8-, 9-, 11-, and 12-peroxy radicals show a tendency to generate their corresponding endoperoxides rather than hydroperoxides. For example, 11-peroxy radical (1) generates endoperoxide (4) [15,16] (Figure 3). The endoperoxides subsequently produce a broad range of reactive intermediates, such as F_2_-IsoPs and MDA. 

Various bioactive aldehydes, such as MDA, 4-hydroxynonenal (4-HNE), and acrolein, are generated by free radical-mediated lipid peroxidation of PUFA, including arachidonic and linoleic acid [7,8,9]. All these aldehydes have been found to play a role in the toxic effects of lipid peroxidation. Aldehyde toxicity is based on the alterations of several cell functions, which mostly depend on the formation of covalent adducts with cellular proteins [17]. HNE forms adducts with three different amino acyl side chains, namely Cys, His, and Lys residues, via Michael addition either to thiol (−SH) or to amino (−NH_2_) groups [18]. MDA has been reported to react in vivo with protein-bound lysine residues to form dihydropyridine (DHP)-type adducts including DHP-lysine [(S)-2-amino-6-(3,5-diformyl-4-methyl-4H-pyridin-1-yl)-hexanoic acid [19,20].

The presence of aldehyde-protein adducts has been demonstrated in a wide range of physiological and pathological conditions. It has been reported that the overproduction of bioactive aldehydes results in protein carbonylation associated with a spectrum of disorders including atherosclerosis, diabetes, neurodegenerative diseases and liver disease [9]. For example, MDA-protein adducts have been detected in Apo B fractions of oxidized low-density lipoproteins (LDL) in atherosclerotic lesions [18,21]. Therefore, aldehyde-protein adducts, which result from lipid peroxidation, are useful biomarkers of disease risk and progression.

The assay methods for lipid peroxidation can be divided into two categories: (i) assays based on measurements of the concentrations of lipid peroxides themselves; and (ii) assays based on determination of the end products of lipid peroxidation, such as MDA, isoprostanes, and DHP-lysine.

## 3. Assay of Lipid Peroxidation

### 3.1. Isoprostanes

**Figure 4:**
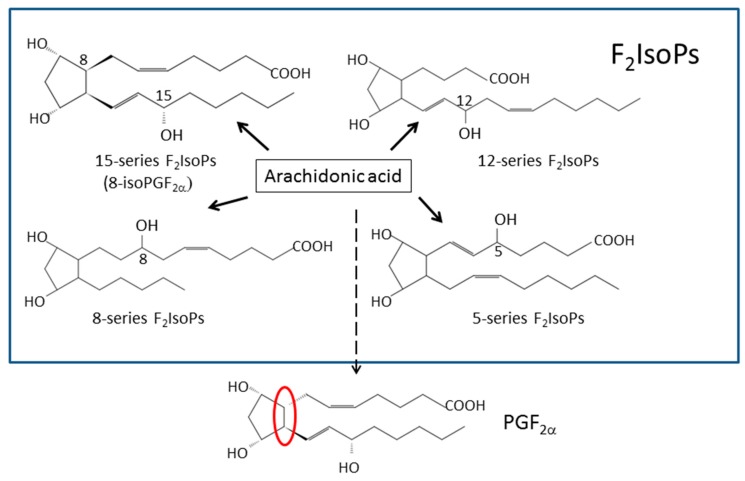
Formation of four classes of F_2_-isoprostanes (IsoPs) from arachidonic acid. 15-series, 5-series, 8-series, and 12-series F_2_-IsoPs are produced from 11-peroxy radicals, 9-peroxy radicals, 12-peroxy radicals, and 8-peroxy radicals, respectively. 8-IsoPGF_2_α is classified into 15-series F2-IsoPs. PGF_2_α is synthesized from arachidonic acid via a cyclooxygenase (COX)-dependent pathway. The structural difference between PGF_2_α and the 15-series F_2-_IsoPs is that the former is an optically pure compound, whereas IsoPs are racemic. The structural distinction from PGF_2_α and 8-isoPGF_2_α is that PGF_2_α contains side chains that are oriented *trans* to the prostane ring (indicated in the red circle).

The isoprostanes (IsoPs) are bioactive prostaglandin-like compounds formed in vivo via a nonenzymatic mechanism involving the free radical-initiated peroxidation of arachidonic acid. A specific class of IsoPs, the F_2_-IsoPs, are measured as indices of endogenous oxidative stress. 8-, 9-, 11-, and 12-peroxy radicals of arachidonic acid are predicted to lead the generation of the four classes of F_2_-IsoPs, respectively (Figure 4, see also Figure 2 and Figure 3). Although various assays for individual IsoPs have been developed [22,23,24,25,26], 8-isoprostaglandin F_2_α (8-isoPGF_2_α, also known as 8-epi-PGF_2_α or 8-isoprostane) is the most commonly used biomarker for the assessment of oxidative stress. As depicted in Figure 4, 8-isoPGF_2_α has a different configuration at C8 chiral carbon, compared with PGF_2_α.

8-isoPGF_2_α can be assessed by either GC/MS or immunological assays. GC/MS is the gold standard for the analysis of IsoPs, however, ELISA is widely used for the measurement of 8-isoPGF_2_α, because ELISA kits have been developed for rapid detection and quantification of 8-isoPGF_2_α. Several studies compared ELISA assays to the GC methods for the measurement of 8-isoPGF_2_α. One comparison study reported that the concentrations of 8-isoPGF_2_α determined with ELISA were higher than those determined by GC-MS [27], suggesting that GC-MS and immunological assays do not measure the same compounds. It is likely that GC/MS analysis measures only a single compound, whereas the antibodies used in ELISA cross react with some of the other F_2_-IsoPs or any of their potential metabolites. Because 8-isoPGF_2_α is a relatively minor F_2_-IsoPs in urine, cross-reactivity of the antibodies needs to be checked carefully [24,26]. However, the cross-activity may be useful for evaluation of oxidative stress in vivo. Immunological measurement of both the parent molecules and their metabolites is more representative of the oxidative reactions than the measurement of a short-lived parent molecule. It is generally recognized that both GC/MS and ELISA methods provide more reliable assessments of lipid peroxidation than any other test in healthy individuals and in those with different types of diseases, including diabetes [28,29]. 

F_2_-IsoPs are predominantly present in the phospholipid domains of cell membranes and plasma lipoproteins. They are significantly higher in high-density lipoprotein (HDL) compared with LDL or very low-density lipoprotein (VLDL) [30,31]. Furthermore, HDL_3_ particles contain high levels of F_2_-IsoPs compared to HDL_2_. F_2_-IsoPs are released by phospholipase A_2_ or platelet activating factor acetylhydrolase (PAF-AH), which hydrolyzes esterified F_2_-IsoPs from phospholipids. The latter enzyme seems more important in human plasma, since plasma samples from patients lacking PAF-AH do not release F_2_-IsoPs from esterified precursors [32]. Hence, F_2_-IsoPs exist in two forms in plasma, free (unesterified) or bound (esterified) to phospholipids in HDL.

Circulating F_2_-IsoPs are partly metabolized and finally are secreted in urine through filtration in the glomerular apparatus of the kidneys. Measurement of urinary rather than plasma 8-isoPGF_2_α has been proposed as a better index of oxidative stress, due to the ex vivo artifactual formation of isoprostanes resulting from auto-oxidation of lipids in plasma [29,33]. However, others prefer determination of plasma total F_2_-IsoPs level, mainly due to inter-individual variations in the rapid metabolism of these compounds [34]. 

8-isoPGF_2_α levels fluctuate during the day under oxidative states. Therefore, it is recommended to measure 8-isoPGF_2_α in 12- or 24-h urinary samples. However, the majority of studies use spot analysis of urinary 8-isoPGF_2_α rather than 24-h collection to avoid the need to check compliance of participants. Importantly, variations in urine flow rate could also affect the assessment of urinary 8-isoPGF_2_α. Urinary creatinine is frequently used for normalization of 8-isoPGF_2_α levels, based on the implicit assumption that creatinine excretion is constant across and within individuals. This is a reasonable approach for individuals as creatinine is continually produced in skeletal muscles and excreted in the urine at a stable rate. However, any variability in creatinine excretion may complicate the determination of urinary 8-isoPGF_2_α levels. Because creatinine serves as a biomarker of glomerular filtration rate, impairment of renal function could affect this commonly applied dilution calculation [35].

Isoprostanes are primarily generated in a free radical-dependent and non-enzymatic fashion [36], but can also be generated by activation of enzymes, such as cyclooxygenase-2 (COX-2). Indeed, COX-2 contributes to the generation of 8-isoPGF_2_α in monocytes and in vascular cells in the pulmonary circulation in various pathological conditions [37,38]. It has been reported that aspirin treatment did not suppress the high levels of urinary 8-isoPGF_2_α found in smokers [39]. Furthermore, the use of non-selective COX inhibitors in rats did not lower the high levels of isoprostanes induced by systemic application of CCl_4_, a known inducer of free radical formation [33]. Therefore, it is most likely that isoprostanes are primarily a product of non-enzymatic lipid modifications. Although the relative contribution of enzymatic and non-enzymatic pathways in different pathophysiological conditions to the levels of plasma and urinary 8-isoPGF_2_α is not clear, 8-isoPGF_2_α is considered a good biomarker for the assessment of oxidative stress. 

### 3.2. Malondialdehyde (MDA)

MDA is one of the most commonly used biomarkers for lipid peroxidation. Its plasma levels correlate closely with plasma 8-isoPGF_2_α levels [40]. This aldehyde is an end product of enzyme- and free radical-catalyzed peroxidation of a variety of PUFAs. The amount of MDA is far much more than that of 8-isoPGF_2_α at a molecular ratio level, that is, 500:1 [16,40].

Since MDA exhibits high reactivity and ability to form adducts with many biological molecules, the majority of MDA is bound to DNA and amino acid moieties in proteins. Circulating MDA exists primarily in two forms, free (unbound) and covalently bound to various biomolecules, such as proteins, nucleic acids, lipoproteins, and soluble amino acids. It is important to emphasize whether the free (unbound) or total (bound and unbound) MDA levels are considered. Since a large percentage of circulating MDA is bound to plasma proteins [16], and only small amounts of free MDA are present in biological samples, total MDA level is easier to be measured. Hydrolysis of the protein-bound MDA fraction can be achieved by either treatment of the sample with an alkaline solution or with an acid solution [41].

Determination of MDA levels has historically relied on a reaction with thiobarbituric acid (TBA) to generate the products known as “thiobarbituric acid reactive substances” (TBARS) that can be measured by colorimetry (532 nm) or fluorimetry (excitation at 532 nm and emission at 553 nm). Since the TBARS test is an easy and cost-effective method, it is widely used for routine analysis of MDA in clinical laboratories. However, the use of TBARS test is not advisable due to the lack of sensitivity and specificity. The reason for this advice is the possible reaction of TBA with a variety of chemically reactive carbonyl groups–containing compounds, such as sugars, amino acids, bilirubin, and albumin, producing interference in colorimetric and fluorimetric MDA measurement. The specificity of the TBARS assay is considerably improved by separating MDA-TBA adduct from TBA adducts of other TBARS substances by HPLC [16,42,43]. Measurement of total plasma MDA by HPLC has indicated that MDA levels quantified by the TBARS test are higher than those obtained from HPLC techniques, indicating that spectrophotometric techniques of TBARS are not as specific as the HPLC techniques [44].

Due to the low specificity of TBA reaction, derivatization reagents other than TBA have been used for the analysis of MDA by HPLC, GC-MS, GC-MS/MS and LC-MS/MS. For instance, dinitrophenyl hydrazine (DNPH), a hydrazine-based reagent, has been used for the derivatization, and the resulting DNPH derivatives of MDA have been analyzed in HPLC by utilizing their spectrophotometric properties [45]. Derivatization reagents, such as 3-nitrophenyl hydrazine, have been used in the LC-MS/MS measurement of MDA in human plasma [46]. Another group measured circulating MDA after pentafluorobenzyl bromide derivatization using GC-MS and GC-MS/MS [47]. In that study, the authors reported no circadian rhythm for circulating MDA and urinary 8-isoPGF2α in humans, suggesting that the timing of sample collection may have little influence on quantitative determination of these oxidative stress biomarkers. 

Zelzer et al. [48] measured total MDA levels using the GC-MS method after derivatization of MDA with DNPH. In parallel, total MDA levels were determined by reaction with TBA followed by HPLC (TBA/HPLC) analysis and fluorometric detection. The results showed close correlation between total MDA levels measured by the GC-MS and the TBA/HPLC, although the latter analyses tended to show higher MDA levels, indicating possible formation of non-specific products of the TBA reaction. The GC- and LC-based assays combined with the technology of MS are considered to be more suitable for accurate measurement of MDA. However, it is still not clear whether many MDA species, free (unbound) and bound MDA molecules, can equally react with the derivatization reagents and contribute to the analytical outcome. MDA concentrations obtained by different reagents and reaction conditions are likely to deviate, thus excluding dependable comparison [16]. 

In addition to the analytical method itself, special precautions are required at the pre-analysis stage. Artefactual formation of MDA depends on blood sampling, storage conditions, and storage time. With regard to blood sampling, the lowest MDA concentrations were measured in freshly generated serum and heparinized plasma samples, compared with stored samples, suggesting that blood MDA levels should be measured in serum or heparinized plasma samples as soon as possible after sampling [47]. Tsikas [16] recommended the description of pre-analytical conditions, such as storage condition and storage time, in any relevant scientific report.

### 3.3. d-ROMs Test

The diacron reactive oxygen metabolites (d-ROMs) test was designed to quantify the peroxidation products themselves rather than their end-products. This test is based on the principle that transition metal ions released from serum proteins under acidic conditions (pH 4.8) stimulate the conversion of hydroperoxides to alkoxyl (R–O•) and peroxyl (R–OO•) radicals (reactions 1 and 2), which subsequently react with the chromogen *N,N*′-dimethyl-p-phenylenediamine (reaction 3) [49]. The resultant colored product can be measured at 505 nm.

R–OOH + Fe^2+^ → R–O• + Fe^3+^ + OH^−^ (1)

R–OOH + Fe^3+^ → R–OO• + Fe^2+^ + H^+^ (2)

R–O• + A–NH_2_ → R–O^−^ + [A–NH_2_•]^+^ and R–OO• + A–NH_2_ → R–OO^−^ + [A–NH_2_•]^+^ (3)

where A–NH_2_:Chromogen.

The alkoxyl or peroxyl radicals can also react with the hydrocarbon part of an unsaturated lipid molecule (reaction 4), followed by the reaction of radicals (R•) with oxygen molecules leading to the generation of R–OO• (reaction 5). Once R–OO• is generated, a propagation of chain reactions to produce R–OOH will take place. In actuality, the d-ROMs test measures not only pre-existing lipid peroxides but also those generated by a radical chain reaction.

R–H + R–O• (R–OO•) → R• + ROH (R–OOH)(4)

R• + O_2_ → ROO• (5)

Essentially all circulating plasma iron is bound to transferrin, which is a monomeric serum glycoprotein (~80,000 daltons) that binds Fe^3+^. Thus, lipid peroxide is measured in the d-ROMs test in the presence of transferrin-derived Fe^3+^. Due to this requirement of iron ions, serum and heparinized plasma, but not EDTA plasma or urine, are used in the d-ROMs test. Normal serum iron levels vary between individuals (ca. 60 to 170 µg/dL) and also throughout the day in the same individual. Furthermore, iron deficiency, iron overload, and inflammation-related anemia are the commonest iron-related disorders. Therefore, the individual difference in blood iron levels may affect the measurement accuracy of the d-ROMs test. Nevertheless, the d-ROMs test has been utilized in many clinical trials for measurement of oxidative stress, because it is a simple test providing an easy way of measurement and detection of hydroperoxides. 

### 3.4. Fe-ROMs Test

As described above, the d-ROMs test requires transferrin-derived Fe^3+^. It also requires reagents supplied by the manufacture. However, the exact information on the reagents is not available. To overcome these obstacles, a d-ROMs-modified method, termed the “Fe-ROMs test”, was developed, in which iron ions are exogenously added to the reaction mixture [50]. In the Fe-ROMs test, 0.1 M acetate buffer (pH 4.8) containing 100 mM *N,N*′-diethyl-p-phenylenediamine and 100 µM Fe^2+^ or Fe^3+^ is first added into each of 96 wells of the microplate, followed by the addition of serum or plasma samples and measurement of absorbance at 505 nm. Absorbance linearly increases in the presence of either Fe^2+^ or Fe^3+^. The rates of increase in absorbance are much larger in Fe^2+^-supplemented reaction mixtures than in Fe^3+^-supplemented ones. The difference between Fe^2+^ and Fe^3+^ may be explained by the finding that Reaction 2 (described in Section 3.3) progresses more slowly than Reaction 1 [51]. 

It has been demonstrated that both Fe^2+^ and Fe^3+^ enhance the rates of increase in absorbance in a concentration-dependent manner up to 100 µM [50]. Fe concentrations in a reaction mixture for the d-ROMs test vary greatly according to the samples used, but they are usually expected not to exceed 1 µM. Because the Fe-ROMs test was performed in the presence of Fe concentrations far higher than 1 µM, usually 100 µM [50], this test can exclude the assay variation related to differences in blood iron levels among individuals. However, previous studies showed a strong correlation between the concentrations measured by the d-ROMs and Fe-ROMs tests in subjects with acute febrile disease, although it tended to be non-linear [50]. Further studies are needed to compare the concentrations measured by the two techniques in a large population, particularly in subjects with low and high levels of serum iron ions.

Lipid hydroperoxides in serum lipoproteins are the most likely entities measured by the Fe-ROMs test. We isolated the HDL from the other lipoproteins with the dextran sulfate-Mg^2+^ precipitation procedure and found that the hydroperoxides in serum are mostly included in the HDL fraction, not in the LDL/VLDL [50]. Therefore, the amount of total hydroperoxide (total ox) in the samples, which was determined by the Fe-ROMs test, represents the amount of oxidized HDL (oxHDL), at least in healthy subjects. However, lipoproteins other than HDL, such as LDL, may carry a substantial amount of hydroperoxides in subjects with cardiovascular disease or low antioxidant intake, and in heavy smokers. Therefore, we recommend using plasma or serum samples in a primary assay for the determination of oxHDL, and if necessary, supernatants after dextran sulfate-Mg^2+^ precipitation in a secondary assay. 

The Fe-ROMs test can quantify both the oxidative stress status and oxidized HDL levels. HDL is atheroprotective based on its ability to reversely transport cholesterol and remove oxidized lipids from oxidized LDL (ox-LDL) [52,53,54]. Since oxidization of HDL weakens or modulates these actions [55,56,57,58], the Fe-ROMs test is a suitable technique for the assessment of HDL functionality in daily clinical practice. Erythrocytes are vulnerable to oxidative damage due to the high cell concentration of oxygen and hemoglobin. Based on their PUFA chain-rich plasma membrane, erythrocytes are susceptible to lipid oxidation [59]. Nakagawa et al. [60] measured the concentrations of lipid peroxides in total lipids extracted from packed erythrocytes by using HPLC and chemiluminescence. They found significantly higher levels of peroxidised phospholipid were accumulated in the erythrocytes of dementia patients. Recently, we developed a more rapid method that can determine the concentrations of lipid peroxides in whole blood [61]. This method, termed the “Fe-ROMs whole blood test”, is essentially similar to the Fe-ROMs test. In the Fe-ROMs whole blood test, glucose is added to 0.1 M acetate buffer containing 100 mM *N,N*′-diethyl-p-phenylenediamine and 100 μM Fe^2+^ to adjust osmolality. Furthermore, the pH of the reaction mixture is increased from 4.8 to 5.8. After 5 min incubation with whole blood at 37 °C, supernatants or filtrates are obtained by centrifugation or a blood separation device (Leisure Inc., Tokyo, Japan), and their absorbance is measured at 505 nm. The above-mentioned modifications prevent hemolysis and allow evaluation of the state of oxidative stress in whole blood. Our results demonstrated that the peroxides in whole blood of healthy humans are mostly present in plasma but not in the blood cellular components [61]. Further research is required to assess oxidative stress using the whole blood test in subjects with different pathophysiological conditions including dementia.

## 4. Diabetes and Oxidative Stress

### 4.1. Diabetes Complications

Diabetes mellitus (DM) is a metabolic disorder characterized by a high level of blood sugar (hyperglycemia), resulting from defects in insulin secretion or insulin action or both. DM is strongly associated with both microvascular complications (diabetic nephropathy, neuropathy, and retinopathy) and macrovascular complications (coronary artery disease, peripheral arterial disease, and stroke) [62]. Forty years ago, the Framingham study reported that the risk of a coronary heart disease event was 2- to 3-fold higher in patients with DM than nondiabetic patients [63]. Subsequent studies confirmed the importance of long-term glycemic control in the prediction of not only microvascular disease but also of macrovascular complications. Moreover, the Diabetes Control and Complications Trial/Epidemiology of Diabetes Interventions and Complications (DCCT/EDIC) study concluded that improvement of glycemic control over a period of 8 years in patients with type 1 diabetes had significant beneficial effects on microvascular complications and reduced the risks of macrovascular complications [64]. In addition, the UK Prospective Diabetes Study (UKPDS) demonstrated that intensive glycemic control reduced the development of cardiovascular disease (CVD) as well as diabetic microangiopathy during long-term follow up of patients with type 2 diabetes (T2DM) [65]. Interestingly, the contribution of hyperglycemia to CVD risk is much greater in type 1 than in T2DM due to other risk factors present in T2DM patients [66]. For example, a 1% increase in HbA1c is associated with >50% increase in CVD risk in patients with type 1 diabetes compared with 7.5% increase in patients with T2DM [67,68].

### 4.2. Hyperglycemia and Oxidative Stress

Hyperglycemia is associated with massive production of ROS. Several studies have investigated the relationship between biomarkers of oxidative stress and patients with DM and diabetic vasculopathies. For example, patients with either type 1 or T2DM have high levels of 8-isoPGF_2_α [69,70,71,72]. 

Any measure of hyperglycemia that assesses glucose control should reflect blood glucose level and glycemic variability (GV). Various methods are used to quantify blood glucose level and GV. Glycated hemoglobin (HbA1c), glycated albumin, and fasting plasma glucose (FPG) reflect the average blood glucose level, whereas standard deviation (SD), mean amplitude of glycemic excursions (MAGE), and M value are frequently used to assess GV [73].

While HbA_1c_ measures the mean glycemic exposure during the preceding 2 to 3 months, it does not reflect the degree of GV (the frequency and magnitude of glucose excursions) that a patient may experience during a given day [74]. Therefore, HbA_1c_ cannot be used to determine whether abnormal glycemic levels are primarily due to high FPG levels or high postprandial plasma glucose (PPG) levels. Several studies have reported that PPG is associated more with CVD than FPG [75,76,77]. Therefore, GV has been proposed as one of the risk factors for T2DM patients.

Several studies have reported that GV is strongly associated with oxidative stress. Monnier et al. [29] studied the relationship between oxidative stress and hyperglycemia. In their study, hyperglycemia was estimated by MAGE and HbA1c, whereas oxidative stress was assessed by 24-h urinary excretion rates of free 8-isoPGF_2_α using an enzyme immunoassay method. The results showed that the triggering effect on oxidative stress was more specific with GV during the postprandial period than chronic sustained hyperglycemia. One possible explanation of this GV effect is that large glucose excursions during postprandial periods cause the generation of excess ROS that exceeds antioxidant capacity. An alternative explanation is the stronger association of 8-isoPGF_2_α levels with MAGE, compared with HbA1c. HbA1c reflects long-term control of blood glucose, whereas MAGE reflects intra-day mean glucose level. In other words, 8-isoPGF_2_α levels reflect more strongly the effects of oxidative stress in recent days. 

Hypoglycemia is a fact of life for patients with both type 1 and type 2 DM. Intensive diabetes control is beneficial for patients with diabetes, but it increases their risk of hypoglycemia. Hypoglycemia causes pathophysiological effects on the cardiovascular system, such as increases in heart rate and peripheral blood pressure and decreases in central blood pressure [78]. Continuous glucose monitoring such as MAGE has been of great value for a comprehensive approach to circadian changes in blood glucose levels including hypoglycemia. Feldman-Billard et al. [79] reported that hypoglycemia correlated closely with elevation of blood pressure in 12 diabetic patients with hypoglycemic episodes and proposed that the hypoglycemia-induced rise in blood pressure is amplified in patients experiencing frequent and severe hypoglycemia. Therefore, hypertension-mediated oxidative stress may be another explanation for the stronger association of 8-isoPGF_2_α levels with MAGE.

Another study [69] showed that improvement of metabolic control (HbA1c, from 9.96 ± 6.3% to 7.5 ± 1.0%, and FBG, from 306 ± 118 to 159 ± 47 mg/dL, mean ± SD) in non–insulin-dependent DM patients was associated with a significant reduction in 8-isoPGF_2_α levels (from 533 ± 276 to 365 ± 226 pg/mg creatinine). Furthermore, Assaloni et al. [80] reported that administration of mitiglinde, a fast-acting, short-duration, insulin secretagogue, resulted in stimulation of insulin secretion and reduction of postprandial hyperglycemia in T2DM patients. Mitiglinde also decreased plasma levels of nitrotyrosine, MDA, and oxidized LDL. Thus, it appears that therapies that stabilize blood glucose levels also reduce oxidative stress in patients with T2DM. However, another group reported that mitiglinde treatment neither improved the levels of urinary 8-isoPGF_2_α, urinary 8-OHdG, nor plasma ox-LDL, although it improved postprandial hyperglycemia and daily GV [77]. The inconsistency of the results may be due to the different sampling time in the above two studies. In the study of Assaloni et al. [80], mitiglinde was administered at time 0, and blood samples were obtained at 120 min for measurement of nitrotyrosine, MDA, and ox-LDL levels, whereas blood and urine samples were taken after an overnight fast in the latter study [77].

**Figure 5:**
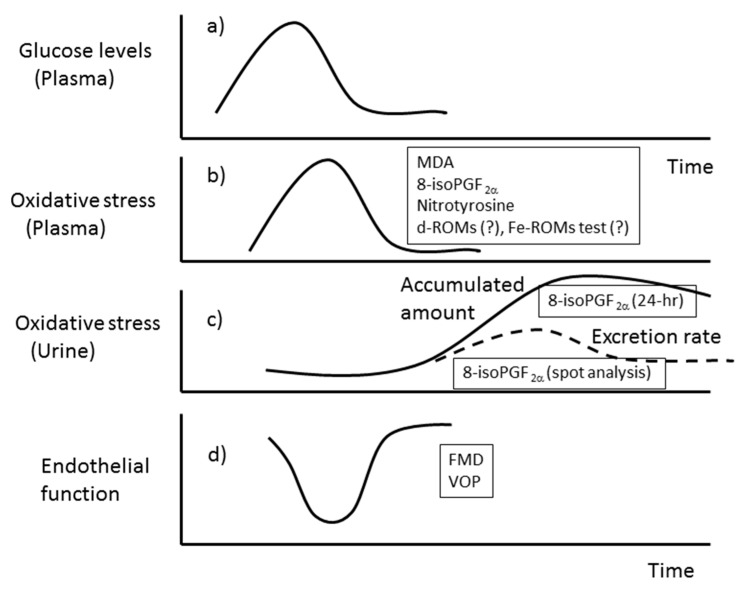
Time course of changes in plasma glucose, oxidative stress, and endothelial function. (**a**) Plasma glucose levels increase after a meal and then return to the baseline. (**b**) Changes in oxidative stress can be assessed by periodic measurement of plasma levels of malondialdehyde (MDA), 8-isoPGF_2_α, or nitrotyrosine after a meal. These markers of oxidative stress increase and return to baseline in a time course manner similar to the changes in plasma glucose level. (**c**) Plasma 8-isoPGF_2_α is gradually excreted into urine. Therefore, the excretion rate is expected to increase and thereafter decline, whereas accumulated urinary 8-isoPGF_2_α reaches a maximum. (**d**) Endothelial function is assessed by venous occlusion plethysmography (VOP) and flow-mediated dilatation (FMD) (FMD and VOP will be explained later in the text). Endothelial function is impaired concomitantly with the increase in plasma glucose levels and oxidative stress.

Several studies have used plasma or/and urine samples for the assessment of oxidative stress. In humans, when [^3^H] 8-isoPGF_2_α was infused into the antecubital vein, 75% of the radioactivity was recovered in urine after 4.5 hrs; the major compound was 2,3-dinor-5,6-dihydro-8-isoPGF_2_α, a metabolite of 8-isoPGF_2_α in humans and rats [34]. Thus, it seems that the turnover of free 8-isoPGF_2_α in blood is rapid, not only through metabolism but also by excretion. The authors of that study recommended tracking 8-isoPGF_2_α levels over a period of time, ideally in both urine and plasma, when studying effects of dietary interventions [34]. The causal relationship between glucose levels and oxidative stress should be explored using plasma samples taken at different times during the study, or pooled urine samples. The schematic diagram shown in Figure 5 will help to understand this notion.

The d-ROMs test has been used as a biomarker of oxidative stress, because it is rapid and cost-effective when used in a clinical setting. Ohara et al. [81] reported that the results of the d-ROMs test correlated significantly with HbA1c (*r* = 0.326; *p* = 0.007), MAGE (*r* = 0.565; *p* < 0.001), and Mean of Daily Differences (MODD) (*r* = 0.488; *p* < 0.001), but not with FPG. Moreover, MAGE and MODD independently correlated with d-ROMs by multivariate analysis. These results add support to the above conclusion that GV is more closely related to oxidative stress than chronic hyperglycemia.

## 5. Oxidative Stress and Endothelial Function in Diabetes

### 5.1. Underlying Mechanism of Endothelial Dysfunction

Arterial atherosclerosis with atherosclerotic changes presents within the walls of the arteries is the main pathological cause of coronary artery disease and peripheral arterial disease. Functional changes in the vascular endothelium, which is a monolayer of endothelial cells lining the interior surface of blood vessels, precede the development of morphological atherosclerotic changes and contribute to lesion development and later clinical complications [82,83]. The mechanisms responsible for the development of this endothelial dysfunction have received much attention.

**Figure 6:**
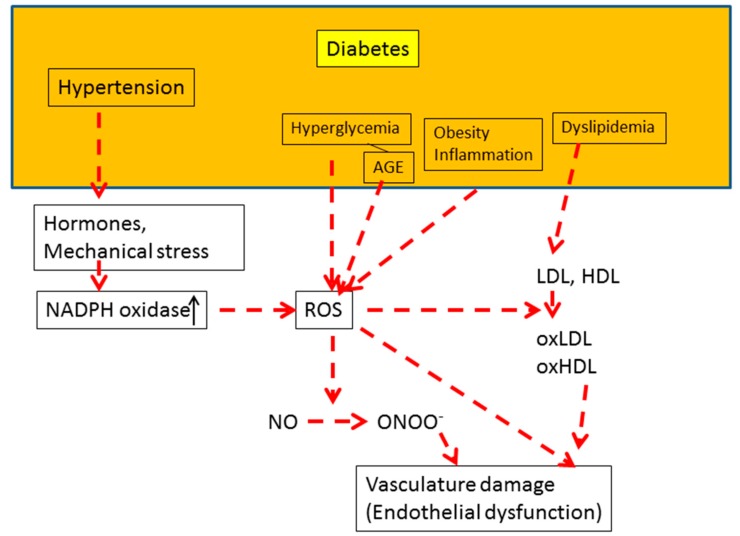
Reactive oxygen species (ROS) are major players in the development of vascular endothelial dysfunction in diabetes. Diabetes mellitus is associated with hyperglycemia, hypertension, obesity, inflammation, and dyslipidemia, all of which lead to vascular damage via ROS-mediated reactions. Details of the ROS-mediated reactions are described in the text (Section 5.3, Section 5.4 and Section 5.5).

T2DM is associated with multiple CVD risk factors, such as dyslipidemia, hyperglycemia, hypertension, inflammation, obesity, and oxidative stress. There is also a close relation between endothelial function and these risk factors. Further, hyperglycemia, hypertension [84], dyslipidemia [85], and obesity [86] are associated with high urinary excretion rates of isoprostanes. Therefore, ROS, which are produced through various different pathways, are considered to be major players in the etiology of vascular endothelial dysfunction in T2DM patients (Figure 6). Based on the presence of various different pathways involved in ROS production, individuals with diabetes or prediabetes may still be at a high risk of development of vascular damage even after excellent glycemic control.

**Figure 7:**
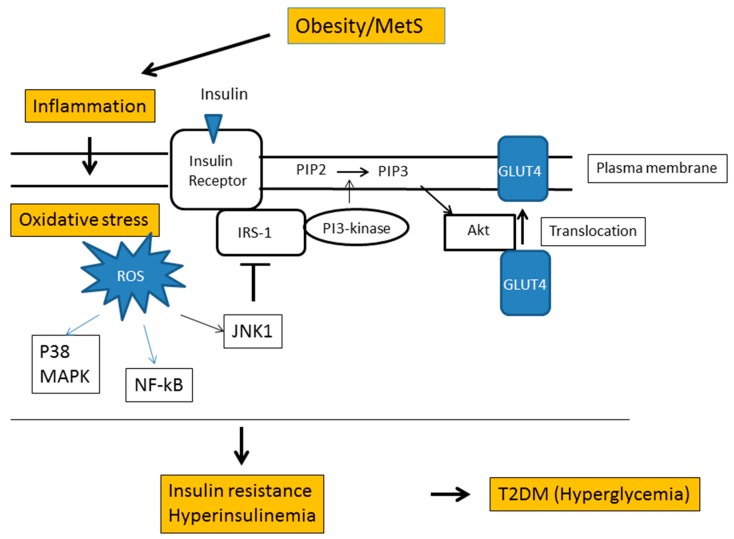
Cause-effect relationship between oxidative stress and type 2 diabetes mellitus (T2DM). Insulin stimulates insulin receptor signaling pathway, including the phosphorylation of insulin receptor substrate-1 (IRS-1), PI3-kinase, and PIP2 and the activation of Akt, eventually leading to the translocation of glucose transporter 4 (GLUT4) to the plasma membrane. Obesity-associated inflammation of the adipose tissue and liver induces macrophage infiltration and increase in pro-inflammatory cytokines and ROS generation. Increased ROS inhibits the insulin receptor signaling pathway, leading to insulin resistance and hyperinsulinemia.

The exact mechanism for the role of oxidative stress in vascular damage and development of diabetes-associated complications is complex. Obesity-associated inflammation causes insulin resistance through several mechanisms (Figure 7) [87]. Increased oxidative stress is one of the risk factors for insulin resistance. Stimulation of the insulin receptor signaling pathway increases cellular uptake of glucose from the blood stream through glucose transporter 4 (GLUT4) translocation to the plasma membrane of skeletal muscle cells and adipocytes. On the other hand, increased ROS levels cause activation of the c-Jun N-terminal kinase (JNK) pathway, thereby leading to increased phosphorylation of serine residue of IRS-1, which no longer functions towards PI3K protein [88]. Therefore, ROS generation impairs GLUT4 translocation and increases intravascular glucose levels. ROS has also been reported to inhibit insulin signal transduction by activation of PKC and nuclear factor kappa B (NF-κB) [88,89]. There is thus a cause-effect relationship between the development of diabetes and oxidative stress. 

### 5.2. Control Mechanisms of Endothelial Function

Endothelial cells play a key role in the control of vascular function and structure by secreting at least three vasodilators: nitric oxide (NO), a prostanoid (mainly prostacyclin), and endothelium-derived hyperpolarizing factor (EDHF). Endothelium-derived NO (eNO) is the major factor involved in the regulation of vascular function. eNO is a multifunctional signaling molecule involved in the control of endothelium-mediated vasodilation, vascular reactivity, platelet activation, thrombus formation, increased permeability, and leukocyte adhesion [90]. eNO is produced by the enzyme endothelial NO synthase (eNOS, also known as NOS3), which is activated in response to fluid shear stress and numerous other factors, such as increased intracellular Ca^2+^, interaction with substrate and co-factors, and protein phosphorylation [91]. eNOS phosphorylation occurs at different sites that can either increase or suppress NO production [92]. Stimuli that activate eNOS phosphorylation include insulin, which plays an important role in vascular homeostasis and maintenance of endothelial function [93]. Inhibition of eNOS by its endogenous inhibitors asymmetric dimethylarginine (ADMA) and monomethylarginine is another important mechanism involved in the regulation of NO synthesis [94].

High ROS levels scavenge bioactive NO through chemical inactivation, forming peroxynitrite, which in turn can uncouple eNOS to form a dysfunctional superoxide-generating enzyme that further contributes to oxidative stress [95]. Thus, ROS reduce the bioavailability of NO as a vasodilator. It was also reported that hyperglycemia is the underlying reason for the attenuated eNOS activity and reduced NO output [96,97]. 

A number of techniques are used to assess endothelial function. For instance, venous occlusion plethysmography (VOP) and flow-mediated dilatation (FMD) are used for the assessment of microvascular and macrovascular endothelial function, respectively [98]. In FMD, an increase in blood flow through the brachial artery produces shear forces on the endothelium and subsequently stimulates endothelial cells to release NO. Reduced vasodilatation following the increase in shear forces reflects impaired NO bioavailability. Arterial stiffness is associated with atherosclerotic risk factors and is an independent predictor of coronary heart disease and stroke [99,100]. The non-invasive brachial-ankle pulse wave velocity (baPWV) is used to assess arterial stiffness, and the cardio-ankle vascular index (CAVI) is an index of the overall stiffness of the artery from the origin of the aorta to the ankle. CAVI is independent of systolic blood pressure measured at the time of its evaluation and has better reproducibility than baPWV [99]. 

### 5.3. Hyperglycemia and Endothelial Function

Postprandial hyperglycemia and hyperlipidemia (abnormal rise in blood glucose and lipid levels that occur after a meal) are considered to induce endothelial dysfunction through ROS production. Several studies support this consideration in both normal and diabetic subjects [95,101,102,103]. For example, vitamin C administration restored endothelium-dependent vasodilation impaired by acute hyperglycemia in healthy humans [102]. 

Ceriello et al. [104] examined the effects of oscillating glucose levels on oxidative stress and endothelial function in healthy subjects and patients with T2DM. They compared three different glycemic profiles over 24 h: (1) glycemia was maintained at 10 mmol/L; or (2) at 15 mmol/L; and (3) 5 and 15 mmol/L every 6 h (oscillating glucose). Oxidative stress was assessed by measuring plasma 3-nitrotyrosine or 24-h urinary excretion rates of free 8-isoPGF_2_α, and endothelial functions were by FMD. Among the above three glycemic profiles, oscillating glucose was more deleterious to endothelial function and caused more intense oxidative stress. Furthermore, the deleterious effects were reversed by concomitant vitamin C infusion, indicating a role of increased oxidative stress in endothelial dysfunction. In their study, large frequent glucose excursions were considered to cause endothelial dysfunction through repetitive overproduction of ROS. Indeed, variability in glycemic control was more deleterious to endothelial cells than constantly high concentrations of glucose in human umbilical vein endothelial cells [105]. Monocyte adhesion to the endothelium may be one of the events leading to endothelial dysfunction and progression of atherosclerosis. Interestingly, repetitive postprandial fluctuation in glucose concentration has been reported to evoke monocyte adhesion to endothelial cells in rats [106]. 

### 5.4. Dyslipidemia and Endothelial Function

T2DM-related dyslipidemia includes high plasma triglyceride (TG) levels, low high-density lipoprotein (HDL)-cholesterol levels, and a preponderance of small, dense low-density lipoprotein (LDL) particles [107]. On the other hand, conventional plasma lipoprotein levels (e.g., total cholesterol, LDL, HDL) are usually normal in type 1 diabetes.

Diabetic dyslipidemia includes both quantitative and qualitative lipoprotein abnormalities. The latter abnormalities include increased glycation and oxidation of LDL, which result from increased glucose concentrations and increased oxidative stress and contribute to the development of atherosclerosis and various vasculopathies [108]. Numerous studies have demonstrated strong correlations between circulating oxidized LDL (ox-LDL) and diabetes [109,110] and that the former plays a key role in the progression of atherosclerosis and diabetes complications. ox-LDL is present on individual apolipoprotein B-100 particles (apoB), and its plasma levels are significant predictors of the presence and extent of carotid and femoral atherosclerosis, development of new lesions, and increased risk of cardiovascular events [111]. 

Low serum HDL level is another major risk factor for coronary disease [112,113] and is predictive of cardiovascular events in statin-treated patients with low LDL-cholesterol levels [114]. Modified LDL, such as ox-LDL, is taken up by macrophages, resulting in their transformation to lipid-loaded foam cells in the intima layer of the arterial wall, which initiates the process of atherosclerosis. HDL or their major protein apoA-I interacts with lipid-loaded macrophages to promote the efflux of excess cholesterol via specific transporters of the ATP binding cassette (ABC) gene family [115], thus helping to protect against the formation of macrophage foam cells. HDL also exerts direct endothelial-protective effects, such as stimulating endothelial cell production of NO and endothelium-dependent vasomotion, exerting antioxidant effects, and promoting endothelial progenitor cell–mediated endothelial repair [116]. 

It has been reported that HDL from patients with T2DM substantially impairs endothelial-protective effects compared with HDL from healthy subjects. The loss of HDL antiatherogenic activity is termed HDL dysfunction. In other words, T2DM represents a state in which HDL becomes dysfunctional [117]. Glycosylation of HDL and apolipoproteins AI (ApoA1), which is known to occur in diabetes, plays a role in the development of HDL dysfunction. Numerous in vitro studies have demonstrated that formation of advanced glycation end-products (AGEs) is associated with impairment of HDL-receptor-mediated cholesterol efflux [118,119]. Another in vivo study also demonstrated that high serum concentrations of AGEs are associated with impairment of the antioxidative capacity of HDL in T2DM patients [120]. However, whether glycation of the HDL protein provides a major effect on HDL dysfunctionality in diabetic patients remains unknown. The relationship between AGEs contents in HDL fraction and the antiatherogenic effects of HDL has not yet been studied in diabetic patients. 

**Figure 8:**
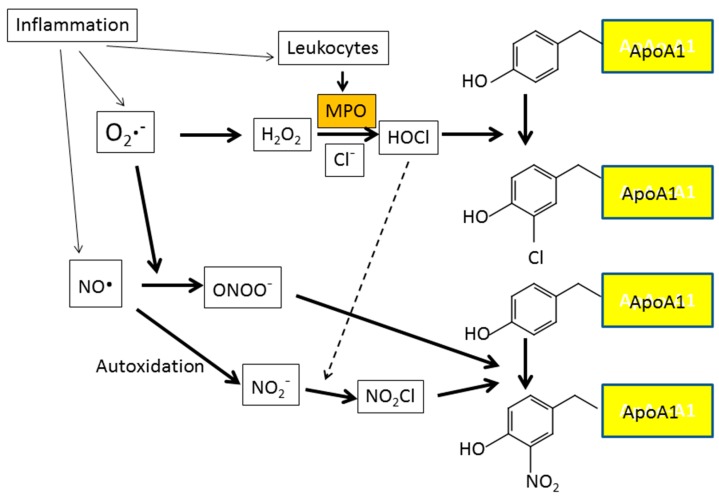
Protein nitration and chlorination caused by the invasion of leukocytes into inflammatory lesions. Myeloperoxidase (MPO), released locally by activated leukocytes, works in concert with ROS and reactive nitrogen species (RNS) and increases protein nitration and chlorination at inflammatory lesions. MPO mediates chlorination and nitration of ApoA1 residues in the pro-inflammatory environment of human atherosclerotic plaque.

Oxidative modification of HDL alters its functions. Oxidation of HDL can be measured by various techniques, one of which is determination of NO_2_Tyr and ClTyr contents in ApoA1. Apo A1 is a primary protein component of HDL and selective target for myeloperoxide (MPO)-catalyzed oxidation (Figure 8). In the study of Zheng et al. [121], HDL was immunoprecipitated from serum and the contents of apoA-I NO_2_Tyr and ClTyr were measured by stable isotope dilution tandem MS. Their results demonstrated impairment of ABCA1-dependent cholesterol efflux from cholesterol-laden macrophages with increased content of apoA-I NO_2_Tyr and ClTyr, suggesting that the oxidative processes catalyzed by MPO participate in the development of “dysfunctional” forms of HDL. MPO-mediated carbamylation of HDL-associated ApoA1 may also render HDL dysfunctional [122]. The carbamyllysine content of lesion-derived HDL was more than 20-fold higher in comparison with ClTyr levels. Because one carbamyllysine residue per apo A-I is sufficient to induce cholesterol accumulation in macrophages, HDL carbamylation is likely to contribute to foam cell formation in atherosclerotic lesions. Thus, tests of HDL function, such as the measurement of HDL-receptor-mediated cholesterol efflux, may be a better predictor of cardiovascular risk than HDL-cholesterol levels.

Hydroperoxidation of lipid components of HDLs is one of the main mechanisms for the loss of HDL-mediated anti-atherosclerotic functions [50,54]. Our group reported that HDL lipid peroxidation is associated with endothelial dysfunction in diabetics [123]. In that study, forearm blood flow (FBF) was measured as an index of endothelial function using VSO, and CAVI was used as an index of arterial stiffness. Oxidative stress was determined from: (1) urine 8-isoPGF_2_α levels; (2) plasma total hydroperoxide (total ox); and (3) the ratio of total ox to HDL (total ox/HDL). Total ox/HDL value was included as an oxidative stress marker, because virtually all of total ox was detected in the HDL fraction, and its level was expected to be dependent on HDL concentrations. Urine 8-isoPGF_2_α and total ox levels were determined by ELISA and the Fe-ROMs test, respectively. The results showed that neither 8-isoPGF_2_α levels nor total ox levels correlated significantly with FBF and CAVI. In contrast, total ox/HDL values correlated with FBF and CAVI, and this correlation was especially strong in T2DM patients with low BMI (<25 kg/m^2^). We concluded that total ox/HDL is a better index of endothelial dysfunction than 8-isoPGF_2_α at least in patients with T2DM. HDL oxidation and the consequent increase in the oxHDL/HDL ratio may play a critical role in endothelial dysfunction in patients with T2DM. 

### 5.5. Hypertension, AGE, and Endothelial Function

A number of studies have shown that hypertension is associated with impairment of endothelial function. Several mechanisms by which hypertension can induce endothelial dysfunction have been reported (Figure 6). Hypertension is associated with the serum concentrations of various vasoconstrictors, such as angiotensin II (Ang II), endothelin-1, and norepinephrine. Ang II effects are mediated via Ang II type 1 and Ang II type 2 receptors, which couple to various signaling molecules, including NADPH oxidase (Nox), which generates ROS [124]. Furthermore, hypertension increases the amount of ADMA, an endogenous eNOS inhibitor, which in turn enhances the production of ROS, causing an imbalance between production of NO and ROS [125]. Therefore, endothelial and vascular smooth muscle NADPH oxidase-dependent ROS are strongly implicated in the hypertension-related vascular dysfunction. On the other hand, ROS have an impact on many systems that influence blood pressure regulation and development of hypertension [126]. Evidence from experimental studies indicates that hypertension is associated in a complex manner with ROS generation and that a cause-and-effect relationship exists between the two [126].

ROS are produced in hyperglycemia through a signaling pathway that involves diacylglycerol (DAG), protein kinase C, and NADPH-oxidase [127]. Under the same environment, it is also induced through mitochondrial respiratory chain enzymes, xanthine oxidases, lipoxygenases, cyclooxygenases, nitric oxide synthases, and peroxidases [128]. In addition, ROS can be generated through the interaction of increased AGEs with RAGE (the receptor for AGEs) in diabetic patients (Figure 6). The AGEs/RAGE interaction activates various signal transduction cascades, including generation of cytosolic and mitochondrial superoxide and activation of transcription factors such as nuclear factor-κB [129]. In cultured human umbilical vein endothelial cells, AGEs/RAGE interaction was reported to induce intracellular generation of ROS through the activation of both NAD(P)H-oxidase and the mitochondrial electron transport system [130]. 

Many experimental studies have indicated that increased formation of AGEs is involved in endothelial dysfunction in diabetes. For example, in one study of patients with T2DM [131], multiple regression analysis that included the following parameters; serum AGEs, age, sex, smoking status, and plasma lipids (total cholesterol, HDL cholesterol, LDL cholesterol, and triglyceride), identified AGEs as the only significant independent determinant of endothelium-dependent vasodilation (*r*^2^ = 0.34, *p* < 0.01). However, the exact underlying mechanism(s) by which AGEs cause endothelial dysfunction remains to be determined. The most probable explanation is that increased serum concentrations of AGEs cause endothelial dysfunction in diabetic subjects via both ROS-dependent and -independent routes. 

## 6. Obesity and Oxidative Stress

Obesity increases the odds of developing many common diabetic complications, including heart disease, retinopathy, dyslipidemia, and hypertension [132]. Weight gain and excess nutrition increase adipose tissue mass and adipocyte size, resulting in damage of adipocytes, initiation of inflammatory processes, and recruitment of macrophages into the adipose tissue [133]. These changes stimulate the entry of pro-inflammatory cytokines, adipokines, and fatty acids into the systemic circulation and the triggering of oxidative stress. The visceral adipose tissue microenvironment was reported to be toxic to the vasculature, with up-regulation of pro-inflammatory and oxidative-stress related mediators that cause severe impairment of arteriolar endothelial vasodilator function [134]. 

Keaney et al. [86] examined 2828 subjects of the Framingham Study to determine the association between oxidative stress and cardiovascular risk factors including obesity. Spot analysis of urinary 8-isoPGF_2_α levels was used for the measurement of oxidative stress. The results showed strong relationships between oxidative stress and smoking, diabetes, and body mass index (BMI). However, quintile BMI analysis revealed no significant association between BMI and urinary 8-isoPGF_2_α levels in men with BMI less than 29.1 kg/m^2^, and a similar finding was noted in women. Furukawa et al. [135] also investigated the association of systemic oxidation stress with obesity. They examined 140 subjects without history of diabetes, cardiovascular diseases, liver diseases, and tobacco abuse. Oxidative stress, as judged by the levels of plasma TBARS and urinary 8-isoPGF_2_α, correlated significantly with BMI (*r* = 0.310 and *r* = 0.432, respectively). However, there was no clear association between obesity and systemic oxidative stress in subjects with BMI less than 30 kg/m^2^. 

It is well recognized that visceral fat accumulation (VFA) is an index of obesity and a major risk factor for obesity-related diseases. Fujita et al. [136] studied the association of systemic oxidative stress, as assessed by urinary 8-isoPGF_2_α, with VFA, subcutaneous fat area, and BMI in 38 non-obese subjects (BMI < 25 kg/m^2^). Among these three obesity indices, only VFA correlated significantly with urinary 8-isoPGF_2_α levels (*r* = 0.728, *p* < 0.001). It thus appears that the BMI value is not a reliable marker of obesity in this context, especially in subjects with relatively low BMI. Instead, VFA is superior to BMI as a predictor of risk of oxidative stress.

Our group also studied the association between VFA and various markers of oxidative stress by performing Pearson correlation analysis on data collected from patients with T2DM [123]. Urinary 8-isoPGF_2_α and oxHDL/HDL (the ratio of oxidized HDL to HDL cholesterol) were used as markers of oxidative stress. Furthermore, oxHDL (amount of hydroperoxide in HDL fraction) was determined by the Fe-ROMs test [50] and used to calculate oxHDL/HDL. While the correlation between 8-isoPGF_2_α levels and VFA was weak, it was stronger between oxHDL/HDL and VFA. It was concluded that the oxHDL/HDL value is a reliable marker for oxidative stress in both clinical setting and experimental studies. On the other hand, urinary 8-isoPGF_2_α did not correlate with VFA. This result seems to contradict the finding of Fujita et al. mentioned above. The different conclusions might be due to the different clinical characteristics of the subjects enrolled in the two studies.

Metabolic syndrome (MetS) is a disorder characterized by the presence of multiple risk factors, including central obesity, hyperglycemia, hypertriglyceridemia, low plasma HDL-cholesterol, and hypertension. A recent study reported that oxidative stress, evaluated by lipid and protein oxidation, is mainly related in patients with obesity to the presence of MetS and to a lesser extent to BMI [137]. The study also showed that nitrosative stress with low NO bioavailability was associated with BMI, independent of the presence of MetS. Therefore, MetS components seem to be an integral part of the process leading to oxidative and nitrosative stress in overweight and obese subjects. It was also reported that systemic lipid peroxidation products are significantly higher and that systemic antioxidant defense capacity was lower in patients with MetS [138]. The risk components of MetS were well controlled in most of the subjects enrolled in the above-mentioned study [123]; the participating subjects might have suffered local oxidative stress but not systemic oxidative stress. Since urinary 8-isoPGF_2_α is acknowledged as a biomarker of systemic oxidative stress [136], there might be no significant correlation between urinary 8-isoPGF_2_α and VFA. On the other hand, oxHDL/HDL could detect even moderate oxidative stress occurring at local sites of vascular injuries. The oxHDL/HDL ratio seems suitable for the detection of local oxidative stress in patients free of multiple risk factors. However, the number of participants (*n* = 28) in our study was too small for full assessment of the relationship between obesity and oxidative stress.

## 7. Inflammation and Oxidative Stress

C-reactive protein (CRP) is an immunomodulatory protein produced by the liver. Acute inflammation induces IL-6-dependent transcription of CRP. CRP is mainly used as a biomarker of significant inflammation. However, the recently-developed high sensitivity assay (hs-CRP) can detect very low levels of CRP, and hence the presence of low-grade chronic inflammation such as vascular inflammation, a known cause of heart disease. Therefore, the hs-CRP assay can be applied to predict CHD in asymptomatic patients, because it can detect the elevation of CRP values several years before the first appearance of clinical features of vasculopathies. 

CRP is also a powerful independent predictor of the development of T2DM in women even after adjustment for obesity, clinical risk factors, and fasting insulin level [139]. Several studies have demonstrated the presence of high levels of IL-6 and CRP among individuals with features of the insulin resistance syndrome and clinically overt T2DM [140,141]. It has also been reported that CRP is significantly associated with increased risk of the development of hypertension [142], suggesting that hypertension is, at least in part, an inflammatory disorder. Pearle et al. [143] reported that CRP is associated with disease progression in patients with osteoarthritis (OA), although other studies have argued against such relationship [144]. 

Oxidative stress and inflammation are closely related pathophysiological processes, one can be easily induced by the other, and both are found in many pathological conditions [145]. Obesity is characterized by chronic low-grade inflammation with permanently increased oxidative stress. Therefore, understanding of the interdependent relation between oxidative stress and inflammation is of great clinical importance. One study showed a statistically significant correlation between CRP and total cholesterol (*R* = 0.19), triglycerides (*R* = 0.29), BMI (*R* = 0.32), FBG (*R* = 0.11), and HDL-cholesterol (*R* = −0.13) (all *p* < 0.0001) [146]. Another group reported a significant correlation between CRP and the Framingham Coronary Heart Disease Risk Score (FCRS), although the correlation between CRP and most individual components of the FCRS was minimal [147]. Furthermore, several studies demonstrated the relationship between inflammation CRP and oxidative stress. For example, in primary hypertension, high levels of inflammatory molecules (CRP and TNF-α) were associated with oxidative stress, as measured by 8-isoPGF_2_α, and with endothelial activation, as analyzed by the soluble forms of ICAM-1 and VCAM-1 [148]. 

**Figure 9:**
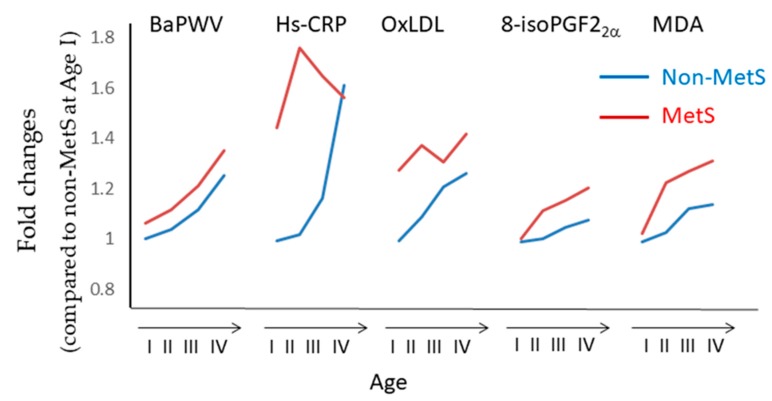
Changes in brachial-ankle pulse wave velocity (baPWV), high-sensitivity C-reactive protein (hs-CRP), and oxidative stress parameters in non-metabolic syndrome (MetS) and MetS subjects. Kim et al. [149] divided subjects into non-MetS and MetS groups. Each group was further subdivided into four groups according to age, the 19-34 (I), 35-44 (II), 45-54 (III) and 55-79 (IV) years groups. BaPWV, hs-CRP, ox-LDL, 8-isoPGF_2_α and MDA levels were measured in non-MetS (I, II, III, and IV) and MetS (I, II, III, and IV) subjects. The line chart was created by using the numerical values provided in [149].

Kim et al. [149] studied the association between baPMW and hs-CRP, components of the MetS, and markers of oxidative stress (plasma oxidized LDL, urinary 8-isoPGF_2_α, and plasma MDA levels) in a total of 4318 subjects, including 807 with the MetS and 3511 without. MDA and 8-isoPGF_2_α levels were measured using TBARS assay kit and ELISA kit, respectively. As summarized in Figure 9, baPMW, hs-CRP, and all markers of oxidative stress increased progressively with age in subjects without MetS, whereas baPMW, 8-isoPGF_2_α, and MDA levels, but not hs-CRP and oxidized LDL, significantly increased with age in subjects with MetS. Therefore, 8-isoPGF_2_α and MDA levels, but not hs-CRP and ox-LDL levels, were associated with arterial stiffness measured by baPMW. Interestingly, both hs-CRP and ox-LDL levels were high even in young subjects with MetS and low baPMW values. MetS is not a single disease, but rather a complex cluster of symptoms. Thus, it requires more detailed analysis focused on individual states.

**Figure 10:**
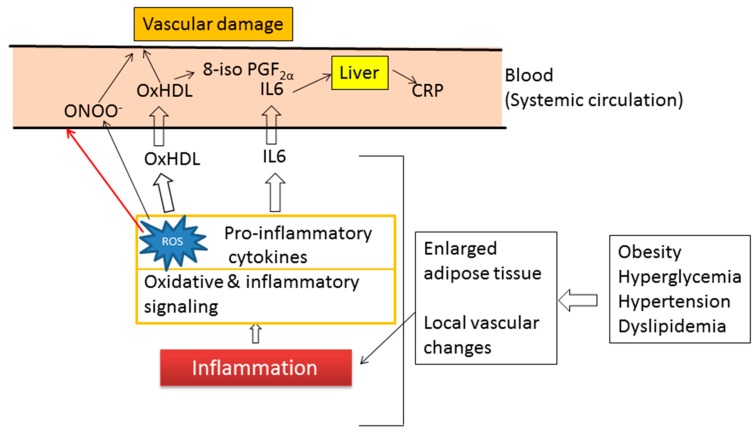
Usefulness of oxidized HDL (oxHDL) as a biomarker of oxidative stress. Local inflammation, which results from increased adipose tissue mass and local vascular changes, induces ROS generation and synthesis and secretion of pro-inflammatory cytokines such as IL-6. IL-6 stimulates hepatic C-reactive protein (CRP) production, whereas increased ROS lead to hydroperoxidation of HDL. Oxidation of HDL impairs its atheroprotective functions, leading to vascular injury. Therefore, oxidative stress (ROS generation and subsequent HDL oxidation) is associated with obesity, inflammation (CRP production), and vascular damage. Oxidative stress can be assessed by the Fe-reactive oxygen metabolites (ROMs) test, and the ratio of oxHDL to HDL is a more reliable biomarker for monitoring oxidative stress than oxHDL. The CRP also causes endothelial cell dysfunction and progression of atherosclerosis, possibly by decreasing nitric oxide synthesis [150].

Recent studies from our laboratories showed a strong correlation between CRP levels and oxidative stress in patients with T2DM [123]. In that study, oxHDL/HDL, but not 8-isoPGF_2_α and oxHDL, correlated significantly with hs-CRP. As summarized in Figure 10, the oxHDL/HDL ratio seems the best predictor of chronic low-grade inflammation associated with increased adipose tissue mass and local vascular changes. 

## 8. Conclusions

DM is a metabolic disorder characterized by hyperglycemia and strongly associated with the development of atherosclerotic plaque. Endothelial dysfunction is one of the initial key steps in atherosclerogenesis in diabetic subjects. Several risk factors, such as hypertension, dyslipidaemia, inflammation, oxidative stress, and AGEs, are associated with atherosclerosis and micro- and macro-vasculopathies. Among these risk factors, oxidative stress is considered to make a large contribution to the formation of atherosclerotic plaques and increased risk of CVD. Despite the accumulated knowledge on the roles of oxidative stress in many serious pathophysiological processes, measurement of ROS is still difficult. Many assay methods are currently available, including measurement of damage of proteins, DNA, RNA, lipids, and other biomolecules. Among them, lipid peroxidation is a useful marker of disease risk and progression. MDA is one of the most commonly used biomarkers for lipid peroxidation. The TBARS test is widely used for routine analysis of MDA in clinical laboratories, because TBARS can be measured by colorimetry (532 nm) or fluorimetry. However, the use of the TBARS test cannot be considered due to the lack of sensitivity and specificity. Urinary 8-isoPGF_2_α is also a good biomarker for the assessment of lipid peroxidation and its level closely correlated with that of MDA. Although most studies use urine spot samples collected after an overnight fast for 8-isoPGF_2_α analysis, it is better to measure 8-isoPGF_2_α in 12- or 24-h urinary samples. The results of the d-ROMs test and measurement of urinary 8-isoPGF_2_α indicate that GV is more closely related to oxidative stress than chronic hyperglycemia. Therefore, GV control, in addition to long-term glycemic control, is important in diabetic patients in order to prevent micro- and macro-vasculopathies. Total-ox/HDL values, but not 8-isoPGF_2_α levels, correlated significantly with endothelial dysfunction and arterial stiffness. Furthermore, the oxHDL/HDL ratio, but not 8-isoPGF_2_α levels, correlated with both VFA and hs-CRP. The total-ox/HDL ratio or oxHDL/HDL ratio, which can be measured by the Fe-ROMs test, is a reliable biomarker for assessment of oxidative stress, endothelial dysfunction, obesity, and chronic inflammation.
